# Efficacy of a Virus-Like Nanoparticle As Treatment for a Chronic Viral Infection Is Hindered by IRAK1 Regulation and Antibody Interference

**DOI:** 10.3389/fimmu.2017.01885

**Published:** 2018-01-04

**Authors:** Karine Chartrand, Marie-Ève Lebel, Esther Tarrab, Pierre Savard, Denis Leclerc, Alain Lamarre

**Affiliations:** ^1^Immunovirology Laboratory, Institut national de la recherche scientifique (INRS), INRS-Institut Armand-Frappier, Laval, Quebec, Canada; ^2^Infectious Disease Research Center, Department of Microbiology, Infectiology and Immunology, Laval University, Quebec City, Quebec, Canada

**Keywords:** plasmacytoid dendritic cells, PapMV, interferon-α, IRAK1, Sca-1, antibodies, lymphocytic choriomeningitis virus clone 13

## Abstract

Although vaccination has been an effective way of preventing infections ever since the eighteenth century, the generation of therapeutic vaccines and immunotherapies is still a work in progress. A number of challenges impede the development of these therapeutic approaches such as safety issues related to the administration of whole pathogens whether attenuated or inactivated. One safe alternative to classical vaccination methods gaining recognition is the use of nanoparticles, whether synthetic or naturally derived. We have recently demonstrated that the papaya mosaic virus (PapMV)-like nanoparticle can be used as a prophylactic vaccine against various viral and bacterial infections through the induction of protective humoral and cellular immune responses. Moreover, PapMV is also very efficient when used as an immune adjuvant in an immunotherapeutic setting at slowing down the growth of aggressive mouse melanoma tumors in a type I interferon (IFN-I)-dependent manner. In the present study, we were interested in exploiting the capacity of PapMV of inducing robust IFN-I production as treatment for the chronic viral infection model lymphocytic choriomeningitis virus (LCMV) clone 13 (Cl13). Treatment of LCMV Cl13-infected mice with two systemic administrations of PapMV was ineffective, as shown by the lack of changes in viral titers and immune response to LCMV following treatment. Moreover, IFN-α production following PapMV administration was almost completely abolished in LCMV-infected mice. To better isolate the mechanisms at play, we determined the influence of a pretreatment with PapMV on secondary PapMV administration, therefore eliminating potential variables emanating from the infection. Pretreatment with PapMV led to the same outcome as an LCMV infection in that IFN-α production following secondary PapMV immunization was abrogated for up to 50 days while immune activation was also dramatically impaired. We showed that two distinct and overlapping mechanisms were responsible for this outcome. While short-term inhibition was partially the result of interleukin-1 receptor-associated kinase 1 degradation, a crucial component of the toll-like receptor 7 signaling pathway, long-term inhibition was mainly due to interference by PapMV-specific antibodies. Thus, we identified a possible pitfall in the use of virus-like particles for the systemic treatment of chronic viral infections and discuss mitigating alternatives to circumvent these potential problems.

## Introduction

Type I interferons (IFN-I), mainly IFN-α and IFN-β, are a family of cytokines with potent antiviral and immunomodulatory properties. The effects of these cytokines on their milieu are complex and affect multiple cells of the immune system by: (i) inducing activation of dendritic cells (DCs) (1, 2); (ii) sustaining activation of CD8^+^ T cells (3, 4); and (iii) inducing differentiation of B cells into antibody secreting cells (5, 6). Thus early IFN-I production is essential for the control of most viral infections such as mouse hepatitis virus (MHV) (7), lymphocytic choriomeningitis virus (LCMV) (8, 9), or simian immunodeficiency virus (SIV) (10). However, whereas early and transient expression of IFN-I controls the infection, prolonged exposure bears detrimental effects to the host’s immune response (10–12). The balancing act between the positive and negative effects of the IFN-I response is observed in the clinical setting as displayed by the well-documented adverse effects of IFN therapy (13, 14). Thus, despite being the standard-of-care against various diseases, emerging therapies now focus on IFN-free alternatives.

Upon viral infection, plasmacytoid DCs (pDCs) are characterized as the main producers of IFN-I [reviewed in Ref. (15, 16)]. This major characteristic is mainly due to their Toll-like receptor (TLR) expression profile. While other innate immune cells express a wide array of TLRs, pDCs mainly express the endosomal TLR7 and TLR9 (16), which bind genetic material typically associated with viral pathogens, such as ssRNA and unmethylated DNA, respectively. These receptors are also capable of recognizing analogs of their natural ligands such as imidazoquinoline and CpG for TLR7 and TLR9, respectively (17). Through their production of IFN-I, pDCs also serve as a bridge between innate and adaptive immune responses as illustrated by their ability to activate natural killer cells (18), other DCs (19, 20) as well as T cells (18, 21). Consequently, upon pDC depletion, mice become highly susceptible to viral infection with MHV (7), herpes simplex virus (22, 23), LCMV (24), vesicular stomatitis virus (18), or the murine cytomegalovirus (18) among others.

By harnessing the central role of pDCs, we have demonstrated the potential for the use of a plant virus-like nanoparticle as a vaccine candidate as well as an adjuvant in various infectious models (25–34). Our platform is based on the papaya mosaic virus (PapMV) nanoparticle that contains a non-replicative synthetic ssRNA rendering it safe for future human use. The synthetic ssRNA found inside the capsid is recognized by the TLR7 of pDCs (31), leading to the production of IFN-α (26, 31, 35), IL-6 (30, 31) along with other cytokines and chemokines. PapMV resultantly activates DCs, macrophages, T cells as well as B cells (26, 28, 31, 35), making this platform versatile in activating the immune system. Furthermore, we have shown that PapMV induces protective immune responses against pathogens when used as a vaccine platform or an adjuvant (26–34, 36) and slows melanoma development when used in an immunotherapeutic setting (37).

Considering that PapMV induces strong IFN-I responses, we sought to evaluate its potential as an immune adjuvant for the treatment for chronic viral infections with the objective of replacing exogenous IFN-α administration with endogenous IFN-α secretion following systemic PapMV delivery. This approach would provide a universally applicable immune stimulatory molecule that could be used against all viral infections without requiring expression of defined viral antigens. We observed that treatment of mice chronically infected with the persistent strain of LCMV (LCMV-Cl13) with PapMV was unable to clear the infection. Moreover, multiple administrations of PapMV induced an immune tolerance as shown by the almost complete abrogation of IFN-α production following secondary PapMV administration. We show that this tolerization is the result of a combination of factors including interleukin-1 receptor-associated kinase 1 (IRAKI) degradation and interference by PapMV-specific antibodies. This information will be crucial for further clinical development of the PapMV platform.

## Materials and Methods

### Ethics Statement

This study was performed in accordance with the Canadian Council on Animal Care guidelines. All *in vivo* experiments were reviewed and approved by the Institut national de la recherche scientifique animal care committee.

### Mice

Female 6- to 10-week-old C57BL/6 mice were purchased from Charles River Laboratories. J_H_T mice were kindly provided by Dr. Rolf Zinkernagel (Zurich University, Switzerland).

### Cells and Virus

Lymphocytic choriomeningitis virus Cl13 was kindly provided by Dr. Rolf Zinkernagel (Zurich University, Switzerland). MC57G fibroblast were cultured in minimal essential medium with Earle’s salt (Wisent, St-Bruno, QC, Canada) containing 5% heat inactivated fetal bovine serum (FBS) (PAA Laboratories, Mississauga, ON, Canada).

### PapMV Nanoparticles

PapMV nanoparticles were provided by Folia Biotech (Quebec, QC, Canada) and were produced as described before (30). Briefly, coat proteins are self assembled *in vitro* around a non-coding ssRNA. Lipopolysaccharide (LPS) contamination was always <50 endotoxin units/mg protein and considered as negligible.

### LCMV Cl13 Infection and Treatment

Mice were infected with 2 × 10^6^ pfu of LCMV Cl13 i.v. and treated with 400 µg of PapMV i.v. on days 3 and 5. Serum was collected 6 h following each treatment to assess IFN-α production. Blood was collected 8 days postinfection (dpi) and mononuclear cells were isolated by density gradient over Ficoll-Paque (GE Healthcare Life Sciences, Mississauga, ON, Canada) and centrifuged at 1,200 rpm for 20 min at room temperature. Cells were collected and washed with PBS then stained for 30 min at 37°C with GP_33–41_ PE tetramers, which were synthesized as previously described (38), to label CD8^+^ T cells specific for the MHC-I gp33 epitope of LCMV. Extracellular staining was performed on unwashed cells for 20 min at 4°C. Following a wash, cells were fixed with fixation buffer (Biolegend, San Diego, CA, USA) for 20 min at room temperature then analyzed by flow cytometry on a BDLSR Fortessa (BD Biosciences, Mississauga, ON, Canada). Spleens collected 30 dpi were disrupted between frosted microscope slides and cells were stained to assess CD8^+^ T cells GP_33–41_^+^ cells as described above. Cells were also incubated with Brefeldin A (BFA) (Sigma, Oakville, ON, Canada) for 5 h at 37°C to inhibit vesicular transport. Spleen cells were then stained for intracellular cytokine production (see below). Blood, spleen, kidney, liver, and brain were also collected 30 dpi to assess viral burden. Organs were mechanically disrupted and supernatants were tittered on MC57G cells by focus forming assay to assess viral burden as previously described (39).

### Immunizations

Mice were injected with 100 or 400 µg of PapMV i.v., 50 µg of imiquimod (R837) (InvivoGen, San Diego, CA, USA), 25 µg of LPS (Sigma), 50 µg of Poly I:C (InvivoGen), or control.

### Organ Processing

Spleen and bones from hind legs were collected at various time points following immunization. Spleens were subjected to digestion with 1 mg/mL of collagenase D (Roche, Mississauga, ON, Canada) for 15 min at 37°C. Femurs, tibias, and iliac crests were flushed and single cell suspensions from both spleen and bone marrow were subjected to red blood cell lysis followed by flow cytometry staining.

### Bone Marrow-Derived Plasmacytoid Dendritic Cells (BMpDCs)

Bone marrow-derived pDCs were prepared by flushing the bone marrow of femurs, tibias and iliac crests, and subjected to red blood cell lysis. Cells were seeded at 2 × 10^6^ cells/mL in RPMI 1640 (Wisent) containing 10% FBS, 100 IU penicillin, 100 µg/ml streptomycin (Wisent), 55 µM β-mercaptoethanol, 1 mM sodium pyruvate, 1× MEM non-essential amino acids, and 10 mM HEPES (Gibco) supplemented with 200 ng/ml of FLT3-L (BioXcell, Lebanon, PA, USA). On days 7–9, cells were stimulated with 100 µg/ml of PapMV, 25 µg/ml of imiquimod (R837) (InvivoGen), 12.5 µg/ml of polyinosinic:polycyticylic acid (Poly I:C) (InvivoGen) or control. On days 8–10, supernatants were frozen at −20°C for IFN-α detection and cells stained for flow cytometry analysis or cell sorting.

### Serum Transfer

Mice were immunized with 100 µg PapMV i.v. on day 0. On day 5 or 25, mice were euthanized and blood was collected by cardiac puncture. Blood was allowed to clot for 30 min at room temperature. Tubes were then centrifuged at 1,500 *g* for 10 min at room temperature. Sera were pooled and injected i.p. to mice whereby the serum from two mice was used to inject one mouse. After 24 h, mice were immunized with 100 µg of PapMV or control i.v. Serum was collected 6 h later to quantify IFN-α and PapMV-specific antibodies and activation of pDCs was assessed in spleen 24 h postimmunization.

### Flow Cytometry

Analysis of surface antigens were performed with the following antibodies and markers: CD3 (145-2C11), CD4 (RM4-5), CD8 (53-6.7), CD44 (IM7), CD62L (MEL-14), PD-1 (29F1A12), Zombie Aqua, CD11b (M1/70), CD11c (N418), CD45R/B220 (RA3-6B2), CD317 (927), CD86 (GL-1), CD69 (H1.2F3), Sca-1 (E13-161.7) (Biolegend). Fc receptors were blocked using a purified anti CD16/32 antibody (2.4G2) (BioXcell). Identification of pDCs was based on their viability (Zombie Aqua^-^) and their surface antigen expression (CD11c^int^, CD11b^lo^, B220^+^, and CD317^+^). For intracellular staining, IFN-γ (XMG1.2), TNF-α (MP6-XT22), IL-2 (JES6-5H4) (Biolegend), IFN-α (RMMA-1) (PBL Assay Science, Piscataway, NJ, USA), IRAK1 (D51G7) as well as isotype control antibodies were used (Cell Signaling Technologies, Beverly, MA, USA) after permeabilization using the Intracellular Staining Permeabilization Wash Buffer 10× and Fixation Buffer following instructions of manufacturer (Biolegend). Data were acquired using BDLSR Fortessa Flow Cytometer (BD Biosciences) and analyzed using the FlowJo software (FlowJo, LLC).

### IFN-α Intracellular Staining

Mice were immunized with 400 µg of PapMV i.v. as described above. Spleens and bone marrows were collected 4 h postimmunization and processed as described above. Cells were then incubated with BFA for 4 h at 37°C followed by IFN-α intracellular staining as described above.

### Cell Sorting

Bone marrow-derived pDCs were stimulated for 24 or 48 h and stained for sorting. Fc receptors were blocked as previously described and pDCs were identified as CD11c^+^B220^+^PDCA1^+^. Cells were collected in FBS then washed twice with cold PBS followed by protein extraction (see below). Sort was performed using a BD FACSJazz (BD Biosciences).

### Immunoblotting

For immunoblotting, cells were harvested and lysed in Triton X-100 lysis buffer [20 mM Tris-HCl pH 8.0, 1% Triton X-100, 10% Glycerol, 150 mM NaCl, protease inhibitor cocktail (Roche)]. Lysates were then loaded on a 10% SDS-PAGE followed by transfer on a polyvinylidene difluoride membranes (BioRad, Mississauga, ON, Canada). Membranes were blocked in 5% dry milk TBS-T (TBS, 0.1% Tween-20) for 2 h at room temperature. Primary antibodies against mouse IRAK1 and β-actin (Cell Signaling Technologies) were diluted in TBS-T and incubated with the membranes o/n at 4°C. Antirabbit IgG HRP (Jackson ImmunoResearch, West Grove, PA, USA) were used as secondary antibodies whereby they were diluted in TBS-T and incubated with the membranes for 1 h at room temperature. Detection was performed with ECL chemiluminescence kit (BioRad).

### ELISA and Multiplex

Interferon-α levels in mice serum or cell culture supernatants were quantified by ELISA according to the manufacturer’s directions (Affymetrix eBiosciences). TNF-α, IL-6, IL-10, IL12p40, IL12p70, IL-9, IL-15, KC, G-CSF, M-CSF, RANTES, MIP-1α, MIP-1β, MIP-2, IP-10, and MCP-1 levels in mice serum were quantified using Milliplex Map Mouse Cytokine/Chemokine Premixed 32 Plex (Millipore, Etobicoke, Canada) according to manufacturer’s directions. Measurement of median fluorescence intensity (MFI) was performed using Bio-plex (Biorad). PapMV-specific antibody titers were determined as described previously (25). Detection of PapMV-specific IgM was performed with peroxidase-conjugated goat antimouse IgM (Jackson Immunoresearch Laboratories).

### Statistical Analysis

Statistical analysis was performed with GraphPad Prism Software (GraphPad Software). Error bars represent SEM. Two-tailed Student’s *t*-test was used and Welch’s correction was applied when needed.

## Results

### Treatment of Chronic LCMV Cl13 Infection with PapMV Does Not Improve Viral Clearance

**Figure 1:**
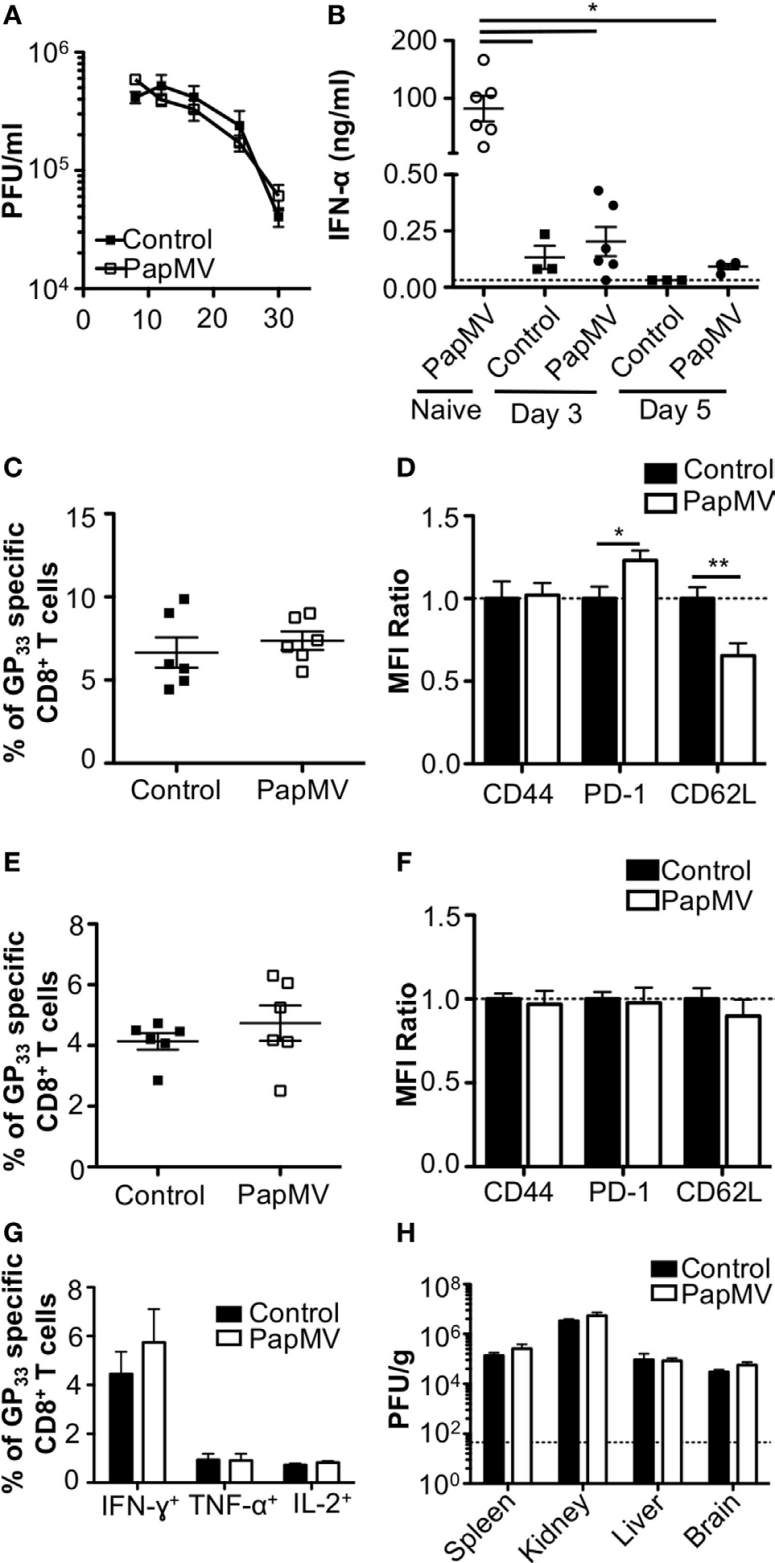
Treatment of an established lymphocytic choriomeningitis virus clone 13 (LCMV Cl13) infection with PapMV does not improve viral clearance. Mice were infected with LCMV Cl13 followed by treatments with PapMV on days 3 and 5 **(A)** Kinetics of viral burden expressed as LCMV Cl13 PFU/ml of blood **(B)** ELISA quantification of interferon (IFN)-α in serum of mice 6 h following treatment with control or PapMV. **(C)** Percentages of GP_33_-specific CD8^+^ T cells in the blood 8 dpi. **(D)** CD44, PD-1, and CD62L expression on CD8^+^ T cells in blood 8 dpi. **(E)** Percentages of GP_33_-specific CD8^+^ T cells in the spleen 30 dpi. **(F)** CD44, PD-1, and CD62L expression on CD8^+^ T cells in the spleen 30 dpi. **(G)** Percentages of CD8^+^ T cells producing IFN-γ, TNF-α, or IL-2 in response to a stimulation with the GP_33–41_ peptide for 5 h. **(H)** Viral loads of LCMV Cl13 in spleen, kidney, liver, and brain 30 dpi. Results are expressed as PFU/g of each organ. For **(D,F)**, results are expressed as a ratio of the sample’s mean fluorescence intensity (MFI) over the average MFI of control samples (*p < 0.05; **p < 0.01; ***p < 0.001) (*n* = 2, three mice per group).

The impact of IFN-α on viral infections in mice was shown to vary according to the kinetics and strength of production during the ongoing infection. Early IFN-α was shown to be essential to the control of the infection (7, 9, 10, 40) while long-term IFN-α was detrimental to the host and favored viral persistence (10–12). In one such study, treatment of LCMV Cl13-infected mice with exogenous IFN-α on days 3 and 5 postinfection resulted in the increase of GP_33–41_-specific CD8^+^ T cells as well as a decrease in viral load 32 dpi (8). This led us to hypothesize that the treatment of a chronic infection such as LCMV Cl13 with PapMV could result in a similar clearance of the virus given the capacity of PapMV to induce potent IFN-α production following immunization (26, 31, 35). We therefore infected mice with LCMV Cl13 and treated them 3 and 5 dpi with 400 µg of PapMV i.v. Blood was collected at various time points to assess viral load, which was not affected by the treatments with PapMV (Figure 1A). To assess the efficiency of the PapMV treatment, serum was collected 6 h following the treatment on days 3 and 5 and IFN-α was quantified by ELISA. Although the administration of 400 µg of PapMV induced strong IFN-α production in naive mice, LCMV Cl13-infected mice barely produced IFN-α at all (Figure 1B) whether assessed after the first treatment on day 3 or the second treatment on day 5. These results indicate that the infection with LCMV Cl13 hinders IFN-α production following PapMV administration. Of note, PapMV not only induces IFN-α in immunized mice but also other cytokines and chemokines such as IL-6, IP-10, and MCP-1 (31, 35), which rely on different sets of signaling pathways than IFN-α for their production. It is therefore possible that immune cells were activated through these auxiliary cytokines following treatment with PapMV despite the absence of detectable IFN-α in the serum. To test this, we assessed activation of CD8^+^ T cells as well as the proportion of GP_33–41_-specific CD8^+^ T cells. The proportion of GP_33–41_-specific CD8^+^ T cells in blood on day 8 postinfection was similar in groups treated with the control or PapMV (Figure 1C) and a similar trend was noticed for the expression of CD44 on CD8^+^ T cells (Figure 1D). We also assessed exhaustion of CD8^+^ T cells by means of PD-1 expression, which was increased in mice treated with PapMV compared to mice treated with control (Figure 1D). Expression of CD62L on CD8^+^ T cells was reduced in PapMV compared to mice treated with control (Figure 1D). Together, these data indicate that the treatment of LCMV Cl13-infected mice with PapMV on days 3 and 5 does not increase the activation of CD8^+^ T cells. However, these treatments seem to increase the exhaustion of CD8^+^ T cells as indicated by the increased expression of the inhibitory receptor PD-1 (41). PapMV treatment did not lead to homing of CD8^+^ T cells in lymphoid organs, as shown by their decrease in CD62L expression, an adhesion molecule involved in the homing of lymphocytes to secondary lymphoid organs. The percentage of GP_33–41_-specific CD8^+^ T cells was not different on day 30 postinfection between control and PapMV-treated group (Figure 1E). As well, the expression of PD-1, CD44, and CD62L was not significantly different between these two groups (Figure 1F). Furthermore, our results revealed that the functionality of GP_33–41_-specific CD8^+^ T cells was not affected by the treatment of LCMV Cl13 infection with PapMV, as shown by the similar percentage of IFN-γ, TNF-α, or IL-2 positive CD8^+^ T cells in the spleen (Figure 1G) between control and PapMV-treated groups. To further confirm that the PapMV treatments had no effect on the clearance of LCMV Cl13, we collected lymphoid and non-lymphoid organs at 30 dpi and assessed the viral load of LCMV Cl13. In the spleen, kidney, liver and brain, both groups displayed similarly elevated LCMV Cl13 titers (Figure 1H), which, along with previous data, indicated that treatment with PapMV was ineffective in clearing an LCMV Cl13 infection. Notably, it has been shown that cells previously infected with chronic viruses such as hepatitis B virus (HBV) (42, 43) or hepatitis C virus (HCV) (44, 45) were unresponsive to further TLR ligand stimulation. Based on our findings, we surmise that a possible TLR tolerance mechanism might be at play after an LCMV Cl13 infection, therefore hindering further activation of immune cells by a subsequent TLR ligand stimulation such as PapMV.

### Pretreatment with PapMV Inhibits Further Effects of Secondary PapMV Administration

**Figure 2:**
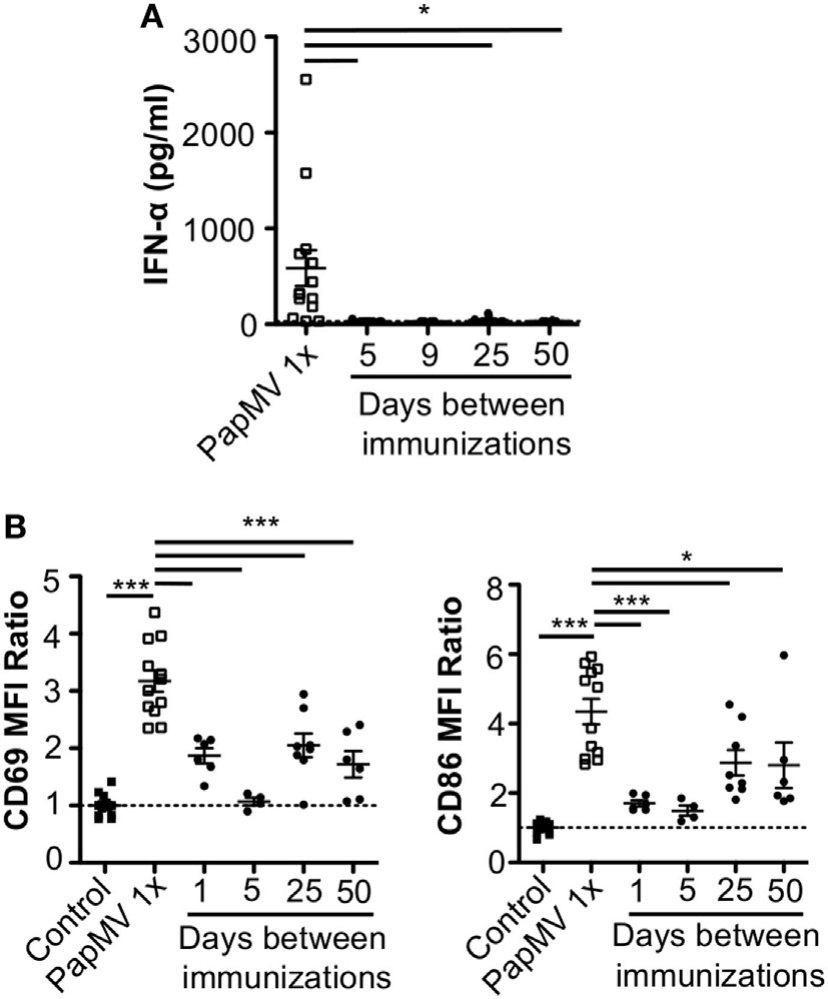
Pretreatment with PapMV prevents plasmacytoid dendritic cells (pDCs) from responding to a subsequent PapMV administration. **(A)** ELISA quantification of interferon (IFN)-α in serum 6 h following immunization with PapMV once (PapMV 1×) or preceded by pretreatment with PapMV at various time intervals. **(B)** CD69 (left) and CD86 (right) expression on splenic pDCs 24 h following immunization with PapMV once (PapMV 1×) or preceded by pretreatment with PapMV at various time intervals. Results are expressed as a ratio of the sample’s mean fluorescence intensity (MFI) over the average MFI of control samples (*p < 0.05; **p < 0.01; ***p < 0.001) (*n* = 2–8, two to four mice per group).

Lymphocytic choriomeningitis virus clone 13 activates immune cells through the TLR7/MyD88 endosomal pathway (46) as well as the RIG-I/Mda5 cytosolic pathway (47). In order to exclude activation and cytokine production caused by engagement of the RIG-I/Mda5 pathway, we decided to move to a PapMV-only based model in which only TLR7 is stimulated. We therefore pretreated mice with PapMV at different time intervals ranging from 1 to 50 days prior to a second immunization with PapMV. Sera were collected 6 h following the second immunization to assess IFN-α production by ELISA. A single administration of PapMV induced strong IFN-α production, as detected in the serum 6 h postimmunization (Figure 2A). When mice were pretreated with 100 µg of PapMV, the production of IFN-α was abolished following a secondary immunization for up to 50 days following pretreatment (Figure 2A). A similar hindrance was observed in the production of TNF-α, IL-6, IL-12p40, IL-12p70, and IL-15, whereas production of IL-10 and IL-9 was enhanced and unaffected, respectively (Figure S1 in Supplementary Material). Similarly, production of various chemokines and growth factors was also suppressed following a second immunization as observed with the production of M-CSF, RANTES, MIP-1α, MIP-1β, IP-10, and MCP-1 whereas production of KC, G-CSF, and MIP-2 was enhanced or unaffected (Figure S1 in Supplementary Material). While there are other cytokines and chemokines that are differentially regulated upon PapMV administration, we focused on IFN-α production based on its wide-ranging use in therapeutic settings (14).

Since pDCs are major producers of IFN-α upon stimulation with a TLR7 ligand, we sought to determine whether the absence of IFN-α was due to a lack of activation of pDCs. Spleens of mice pretreated with 100 µg of PapMV were collected 24 h following the second immunization and activation of pDCs was assessed by flow cytometry. As seen with IFN-α production, a single immunization with PapMV induced upregulation of CD69 and CD86 (Figure 2B) on pDCs. Conversely, when mice were pretreated with PapMV 1–5 days prior to a second immunization, pDCs were unable to respond to the second immunization, as observed by the absence of CD69 or CD86 upregulation (Figure 2B). Here, we found that the expression of activation markers on pDCs on day 1 after the pretreatment was due to remnants of the initial immunization rather than the activation of pDCs following the second immunization (Figure S2 in Supplementary Material). With a 25-day or more lag between the pretreatment and the second immunization, pDCs were activated by the second immunization although to a lesser intensity than mice treated only once with PapMV (Figure 2B). Altogether, these results demonstrate that the administration of PapMV induces a refractory state in pDCs rendering them unable to respond to further PapMV immunizations. In short intervals, this effect is completely inhibitory while for longer intervals the inhibition is only partial, indicating that distinct mechanisms are possibly concomitantly interfering with the response.

### Refractory State Induced by PapMV Pretreatment Affects the Response to Other TLR7 and TLR4 Ligands but Not TLR3

**Figure 3:**
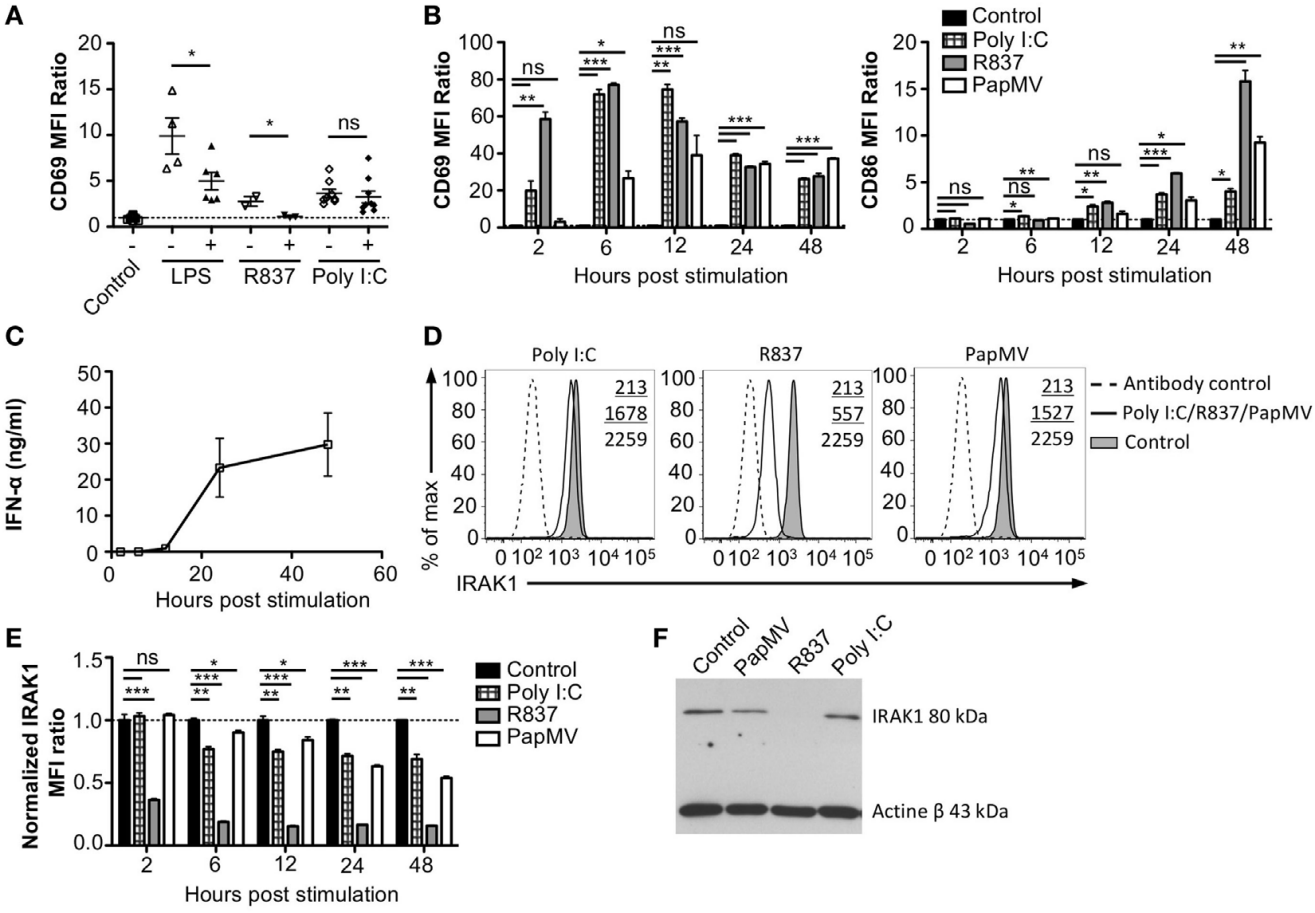
Interleukin-1 receptor-associated kinase 1 (IRAK1) is degraded by PapMV in bone marrow-derived plasmacytoid dendritic cells. **(A)** CD69 expression on splenic plasmacytoid dendritic cells (pDCs) 6 h following the last immunization with the control and R837 and 24 h following immunization with the control, lipopolysaccharide (LPS), or polyinosinic:polycyticylic acid (Poly I:C) once (−) or preceded with a pretreatment with PapMV (+) 5 days prior (*n* = 2–3, two to three mice per group, one representative experiment is shown for R837 samples). **(B)** CD69 (left) and CD86 (right) expression on bone marrow-derived BMpDCs 2–48 h poststimulation with control (black bars), Poly I:C (checkered bars), R837 (gray bars), or PapMV (white bars). **(C)** ELISA quantification of IFN-α in culture supernatant from BMpDCs stimulated 2–48 h with PapMV. **(D)** Representative overlay histograms of IRAK1 expression in BMpDCs following a stimulation of 24 h with control (filled graph), Poly I:C, R837, or PapMV (full line), or secondary antibody staining control (dashed line). **(E)** IRAK1 expression in BMpDCs 2–48 h following stimulation with the control (black bars), Poly I:C (checkered bars), R837 (gray bars), or PapMV (white bars). **(F)** Immunoblot of IRAK1 (top) and actin-β (bottom) of BMpDCs 24 h following stimulation with control, PapMV, R837, or Poly I:C. BMpDCs were sorted to isolate pDCs prior to protein extraction and immunoblotting. **(A,B,E)** Results are expressed as a ratio of the MFI of the sample on the average MFI of control samples. **(A)** (*n* = 2, two to three mice per group) **(B,D,E)** (representative experiment of two to ten experiments, two to three replicates per group) **(C)** (*n* = 2, 2 replicates per time point) (*p < 0.05; **p < 0.01; ***p < 0.001).

To assess whether the refractory state induced by a pretreatment with PapMV affected only further administrations of PapMV, we pretreated mice with PapMV and then challenged them 5 days later with LPS, R837, or Poly I:C, which are ligands for TLR4, TLR7, and TLR3, respectively. We then assessed the expression of CD69 on pDCs (Figure 3A). As expected, mice treated with LPS, R837, or Poly I:C alone showed an upregulation of CD69 on pDCs, indicating activation. When mice were pretreated with PapMV, subsequent immunizations with LPS or R837 were not as efficient in inducing the activation of pDCs as immunizations in naive animals but still showed some degree of CD69 upregulation. These results indicate that the inhibition induced by pretreatment with PapMV not only impacts subsequent immunizations with PapMV but also other TLR7 ligands as well at other TLR pathways, such as TLR4, although the inhibition is less pronounced. Strikingly, unlike the inhibition observed with LPS and R837, administration of PapMV prior to Poly I:C resulted in the expression of CD69, indicative of pDC activation, which was similar in the control and treated groups (Figure 3A). Altogether, these results point toward an inhibitory mechanism induced by PapMV pretreatment that affects TLR7 and TLR4 pathways while the TLR3 pathway remains unaffected.

### Stimulation with PapMV Induces Degradation of IRAK1 in pDCs

Toll-like receptor pathways are not specific to each receptor. Indeed, most of the complexes implicated in the signaling cascades are shared across pathways [reviewed in Ref. (48, 49)]. If PapMV were to affect the TLR7 signaling pathway, other TLR signaling pathways would also be affected, resulting in the cross-inhibition observed when cells are pretreated with PapMV. Indeed, such cross-inhibition was observed in response to stimulations with various TLR ligands such as TLR4 ligands (50–52) TLR7 ligands (52, 53) and TLR9 ligands (51, 53), establishing the presence of homo and hetero tolerance in the TLR signaling pathways. In these studies, one common mechanism reported to be involved in the observed cross tolerance was through the degradation of IRAK1 (52, 54, 55), a kinase implicated in all MyD88-dependant TLR signaling pathways, therefore excluding TLR3, which signals through a MyD88-independent pathway (56). Therefore, we assessed IRAK1 expression in pDCs by flow cytometry and immunoblotting to analyze the regulation of IRAK1 following PapMV stimulation. Given that pDCs account for only 0.2% of all splenocytes, we evaluated the response of pDCs to PapMV *in vitro* using BMpDCs. After differentiation of bone marrow cells with Flt3L for 8 days, cells were stimulated with PapMV. Similar to our observations from *in vivo* splenic pDCs, BMpDCs were readily activated by various TLR ligands including PapMV as shown by the upregulation of CD69, CD86 (Figure 3B) and the accumulation of IFN-α in culture supernatants following stimulation (Figure 3C). Since PapMV activates BMpDCs, we investigated the regulation of IRAK1 following stimulation with various TLR ligands. We first assessed IRAK1 expression by flow cytometry following various incubation periods of BMpDCs with TLR7 ligands, PapMV and R837, or TLR3 ligand, Poly I:C. As expected, R837 induced a strong downregulation of IRAK1 after 24 h in BMpDCs *in vitro* (Figures 3D,E). Surprisingly, stimulation of BMpDCs with Poly I:C induced a small downregulation of IRAK1 starting at 6 h poststimulation. Stimulation of BMpDCs with PapMV induced the degradation of IRAK1 albeit to a lesser extent than R837 and with slower kinetics (Figures 3D,E). To confirm the modulation of IRAK1 expression, we sorted pDCs 24 h poststimulation with PapMV, R837 and Poly I:C and extracted total cellular proteins to evaluate the expression of IRAK1 by immunoblotting. We found that 24 h post-R837-stimulation, the expression of IRAK1 was undetectable (Figure 3F) while stimulation with Poly I:C did not induce any degradation of IRAK1 (Figure 3F). IRAK1 was also degraded following stimulation of BMpDCs with PapMV although the extent of degradation was lower in comparison to that observed with R837 (Figure 3F). When comparing IRAK1 expression ratios obtained by western blot and flow cytometry (Table 1; Figures 3E,F), we noticed similar ratios between the two assays for R837 and PapMV stimulated BMpDCs while the ratios vary for Poly I:C stimulated BMpDCs. Taken together, these results indicate that PapMV induces the degradation of IRAK1 in pDCs, which could in part explain the tolerance observed when mice are pretreated with PapMV.

**Table 1:** Comparison of IRAK1 regulation ratios by flow cytometry and Western blot.

Sample	Flow cytometry ratio	Immunoblot band intensity ratio
Control	1.000	1.000
PapMV	0.631	0.587
R837	0.166	0.026
Polyinosinic:polycyticylic acid	0.716	1.011

### PapMV Induces the Upregulation of Sca-1 on pDCs Despite Its Expression Not Being Associated with IFN-α Production

**Figure 4:**
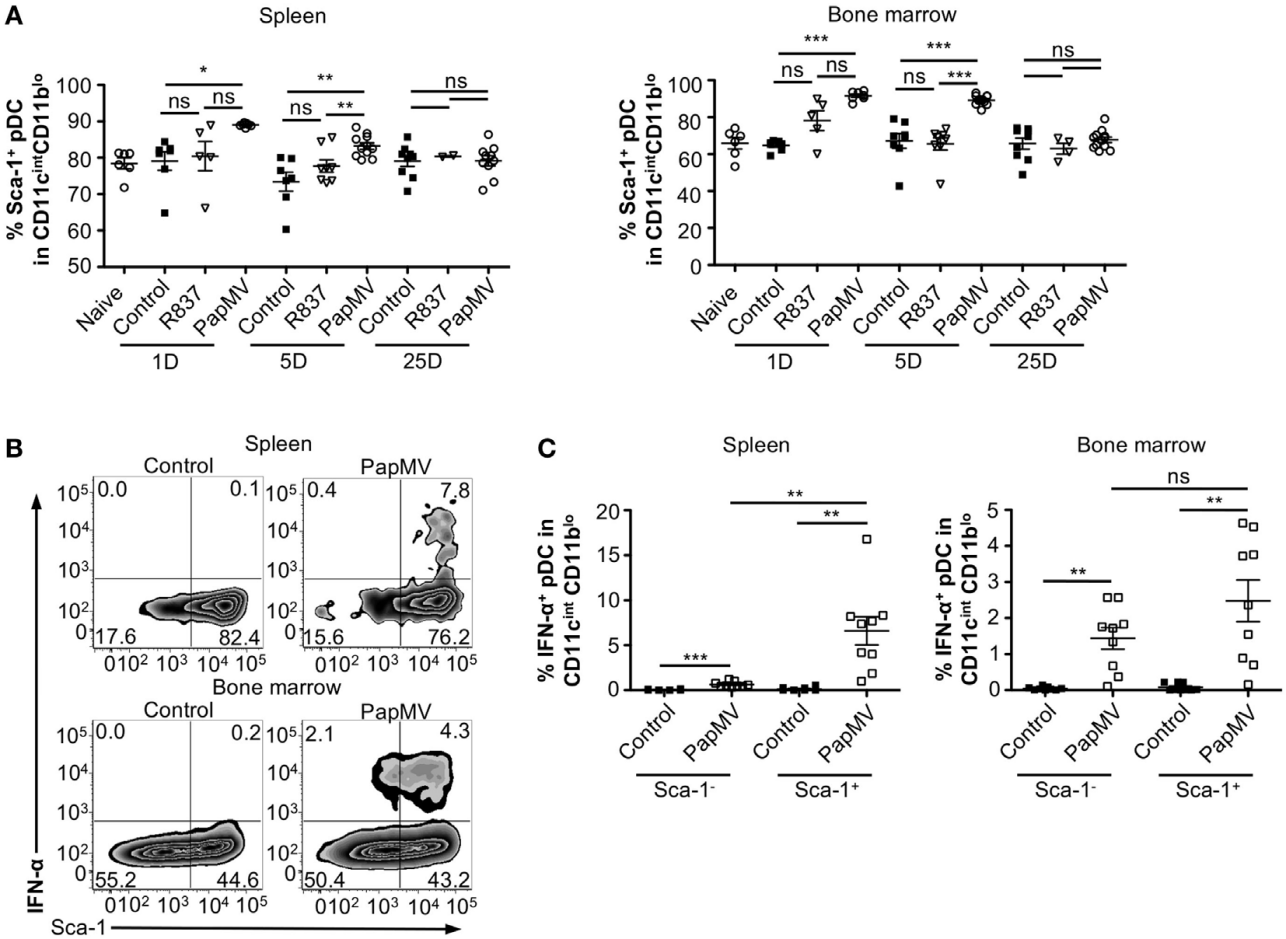
PapMV induces Sca-1 on splenic and bone marrow-derived plasmacytoid dendritic cell (pDCs), despite its expression not being associated with interferon (IFN)-α production. **(A)** Percentages of pDCs from spleen (left) and bone marrow (right) positive for Sca-1 expression 1, 5, and 25 days following an immunization with control (black squares), R837 (open inverted triangles), and PapMV (open circles) (*n* = 2–4, 1–3 mice per group). **(B)** Representative flow cytometry plots of IFN-α production by pDCs according to their Sca-1 expression profile 4 h postimmunization followed by a 4-h incubation with Brefeldin A (BFA). Spleen (top) and bone marrow (bottom) samples are represented. **(C)** Percentages of intracellular IFN-α^+^ pDCs found in the spleen (left) and the bone marrow (right) 4 h following an immunization with control or PapMV followed by 4-h incubation with BFA before intracellular staining (*p < 0.05; **p < 0.01; ***p < 0.001).

Heterogeneity in the pDC population has been described (57–60) although the biological significance of this phenomenon is still largely unknown. Various reports have, however, indicated that two subsets of pDCs expressing different sets of markers were differentially associated with IFN-α production following TLR stimulation (57–59, 61). Among these studies, it has been suggested that expression of Sca-1 could discriminate between IFN-α producing pDCs and those that do not: Sca-1^+^ pDCs were weak producers of IFN-α while Sca-1^−^ pDCs were strong producers (61). Thus, by evaluating the expression of Sca-1 in pDCs, we sought to determine whether it could explain the inhibition in IFN-α production observed with longer periods between PapMV treatments. As previously described (61), pDCs from the spleen are mostly Sca-1^+^ (88.45% Figure 4A; Spleen; Naive) while pDCs from the bone marrow, although still in majority Sca-1^+^ (69.90% Figure 4A; Bone marrow; Naive), display of a larger population of Sca-1^−^ than in the spleen. After an immunization with PapMV, the proportion of pDCs expressing Sca-1 increased to close to 100% in both the spleen and the bone marrow (Figure 4A) and remained elevated for at least 5 days in both organs. The distribution of Sca-1 expression among pDCs returned to naive and control levels by 25 days post-PapMV immunization. To determine which of the Sca-1 expression profile was associated with IFN-α production, we performed IFN-α intracellular staining on pDCs from spleen and bone marrow of mice immunized with 400 µg of PapMV 4 h prior. In the spleen, most IFN-α^+^ pDCs were Sca-1^+^ (Figure 4B). However, in the bone marrow, both Sca-1^−^ and Sca-1^+^ pDCs were expressing IFN-α after a PapMV immunization (Figure 4B). No significant difference was denoted between the two groups in the bone marrow. Niederquell et al. (61) proposed that Sca-1^−^ pDCs could be precursors of Sca-1^+^ pDCs. In this regard, it is possible that the proportion of Sca-1^+^ IFN-α^+^ pDCs are in fact Sca-1^−^ cells that are upregulating Sca-1 in response to stimulation. When intracellular staining for IFN-α was performed 2 and 6 h postimmunization to assess the progression of the IFN-α^+^ population with respect to Sca-1 expression in both spleen and bone marrow, no difference was observed between the three time points (data not shown). Altogether, these results indicate that although PapMV induces expression of Sca-1, IFN-α production does not seem to be associated with Sca-1 expression in this experimental setting.

### Antibodies Are Responsible for Long-term Attenuation of the Response of pDCs to PapMV Immunization

**Figure 5:**
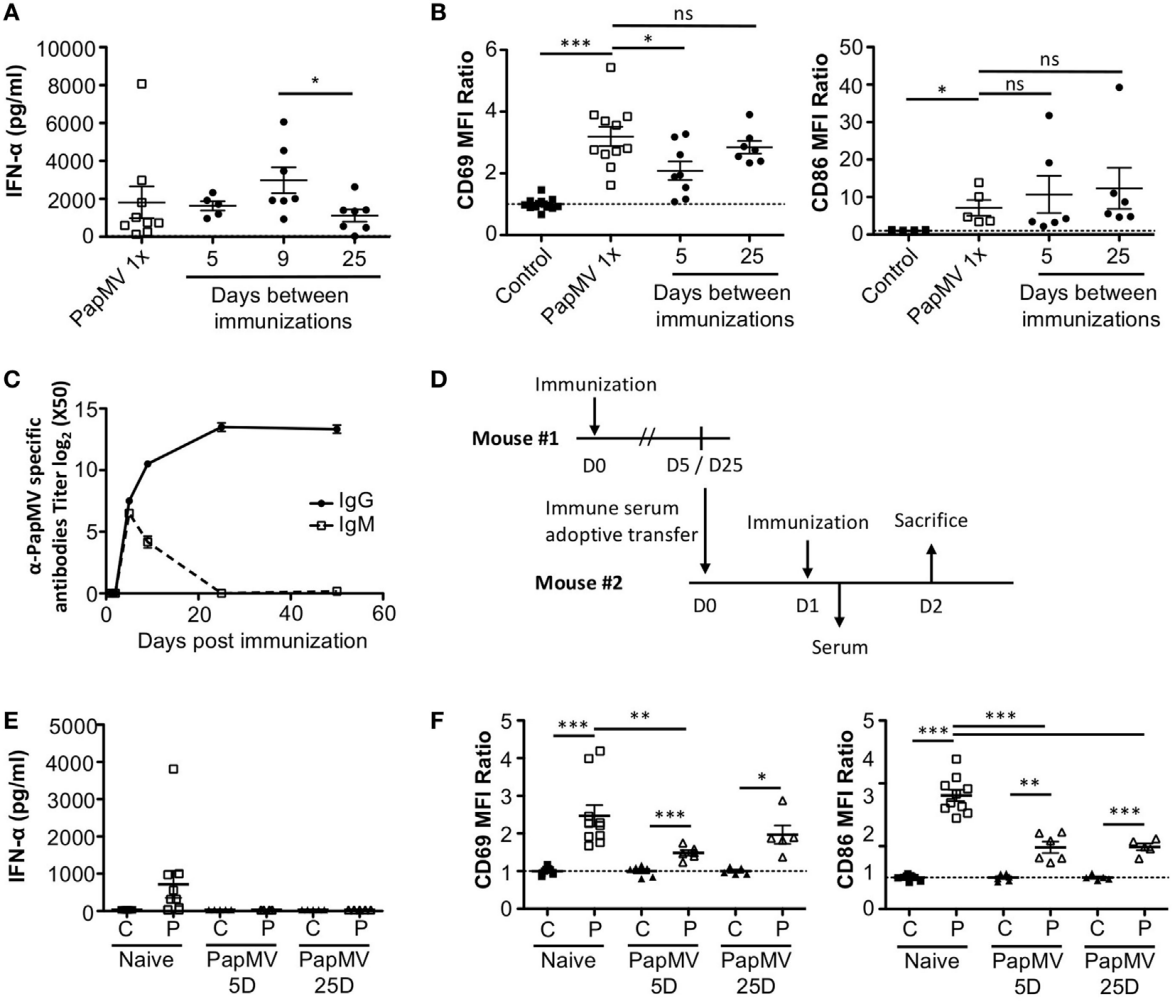
PapMV-specific antibodies are responsible for the long-term attenuation of plasmacytoid dendritic cell (pDC) activation in response to a secondary immunization with PapMV. **(A)** ELISA quantification of interferon (IFN)-α in serum of J_H_T mice 6 h following immunization with PapMV once (PapMV 1×) or preceded by a PapMV pretreatment at various intervals (*n* = 2–5, two to four mice per group). **(B)** CD69 (left) and CD86 (right) expression on splenic pDCs of J_H_T mice 24 h following immunization with control, PapMV once (PapMV 1×) or preceded with a pretreatment with PapMV at various time intervals. **(C)** Kinetics of PapMV-specific IgG and IgM development in C57Bl/6 mouse serum after immunization with PapMV as determined by ELISA. **(D)** Schematic representation of the serum transfer experiments. **(E)** ELISA quantification of IFN-α in serum of C57Bl/6 mice transferred with immune serum 6 h following an immunization with control (C) or PapMV (P). **(F)** CD69 (left) and CD86 (right) expression on splenic pDCs of serum transferred C57Bl/6 mice immunized with control (C) or PapMV (P). **(B,E)** Results are expressed as a ratio of the MFI of the sample of the average MFI of controls (*n* = 3–6, one to three mice per group) (*p < 0.05; **p < 0.01; *** *p* < 0.001).

Our results suggest that inhibition of the response to multiple administrations of PapMV is induced through shared mechanisms between TLR-associated pathways for short periods (Figure 3A) and PapMV-specific components that affect IFN-α production (Figure 2A) as well as pDC activation (Figure 2B) for longer periods. We were therefore interested in assessing the role played by antibodies in the refractory state induced by PapMV pretreatment knowing that they were shown to affect responses to prime-boost vaccine regimens in other systems (62, 63). We determined the antibody-mediated impact of PapMV pretreatment on further PapMV immunizations using J_H_T mice, which lack functional B cells and consequently also lack antibodies (64). As observed in C57Bl/6 mice (Figure 2A), a single immunization with PapMV in J_H_T mice induced the production of IFN-α (Figure 5A) although in a slightly more pronounced fashion. When a PapMV pretreatment was administered 5, 9, or 25 days prior to secondary PapMV immunizations, the production of IFN-α following the second immunization was equivalent to the response of naive animals, which differed significantly from results obtained in C57Bl/6 mice (Figure 5A). Since IFN-α production was not affected by the pretreatment, we examined the expression of CD69 and CD86 on pDCs after multitreatments with PapMV. Similarly to what was observed with IFN-α production, CD69 and CD86 (Figure 5B) expression on pDCs was not significantly different between naive mice and PapMV pretreated mice receiving a PapMV immunization. To confirm that the IFN-α production and pDC activation in J_H_T mice was due to the absence of antibodies and not of B cells, we performed transfer experiments with immune serum. Since two different profiles are observed at day 5 and day 25 in wild-type (WT) mice with respect to the inhibition generated by a PapMV pretreatment, we assessed the kinetics of IgM and IgG production in the serum of mice after a PapMV immunization (Figure 5C). As expected, both IgM and IgG specific for PapMV were found in the serum of WT mice 5 days postimmunization while 25 days postimmunization, only a high IgG titer was detected. Transfers were carried out with serum collected either 5 or 25 days after PapMV administration pooled from matched groups. The serum from the equivalent of two mice was injected into one recipient mouse, which was immunized with PapMV 24 h later (Figure 5D). IFN-α production was assessed in the serum 6 h postimmunization while pDC activation was assessed 24 h postimmunization. Injection of naive serum did not affect the production of IFN-α (Figure 5E compared to Figure 2A) nor did it affect the activation of pDCs as shown by the expression of CD69 and CD86 (Figure 5F compared to Figure 2B). However, when PapMV-immune serum taken at 5 or 25 days was administered to mice, the following immunization with PapMV showed no production of IFN-α (Figure 5E) and a significant decrease in the expression of CD69 and CD86 on pDCs (Figure 5F) although not as much as observed in WT mice. Altogether, these results indicate that PapMV-specific antibodies play a significant role in the long-term inhibition and attenuation of the immune response following a pretreatment with PapMV.

## Discussion

Interferon-α has long been a treatment of choice for chronic viral infections, whether used alone or in combination with other treatments such as ribavirin in the treatment of HCV. Due to its toxicity, the medical community is moving away from IFN-α-based treatments toward alternatives bearing better adverse event profiles. One alternative toward this end would be to induce endogenous IFN-α production by the host instead of administering high doses of exogenous IFN-α. It is with this aim that we used PapMV nanoparticles, which contain a non-coding ssRNA molecule rendering it non-replicative. PapMV induces the production of IFN-α by pDCs without causing any adverse effects when administered systemically and could therefore potentially accelerate the clearance of a persistent LCMV Cl13 infection. This approach would also be potentially applicable to other viral infections, as it would not require expression of virus-specific antigens. We observed that sequential application of PapMV treatment has limitations attributed to regulation of the TLR7 pathway as well as the presence of PapMV-specific antibodies upon the first immunization.

The treatment of an LCMV Cl13 infection with PapMV proved to be inefficient as no changes in viral loads or in LCMV-specific immune responses were observed following treatment (Figure 1). Like PapMV, LCMV Cl13 is also a TLR7 ligand [reviewed in Ref. (15, 16)]. It is therefore possible that the stimulation of TLR7 by LCMV Cl13 induces TLR tolerance similar to that observed when other TLR ligands are used as stimulators (50–53). Further stimulation of the TLR pathways would therefore be inefficient in LCMV-infected mice. Similarly, previous research has shown that stimulation of HBV-infected (42, 43) or HCV-infected (44, 45, 65) human cells with TLR ligands was unable to induce the production of cytokines and activate infected DCs.

Whereas we were unable to clear an LCMV Cl13 infection using PapMV administration, treatment of viral infections with exogenous IFN-α early in the course of the infection has been shown to be efficient in the control of LCMV (8), SIV (10), or RSV (40). Of note, in the successful treatment of an LCMV Cl13 infection with IFN-α, Wang et al. administered the IFN-α5 subtype. However, the IFN-α subtype profile elicited by PapMV has yet to be determined. Thus, the discrepancy observed could be due to a difference in the IFN-α subtype given the disparity in the immunomodulatory effects and antiviral capacities borne by different subtypes (66–68). Furthermore, contrary to direct IFN-α injection, treatment with PapMV requires uptake of the nanoparticle, release and degradation of the ssRNA inside the endosome before IFN-α can be produced following activation of the TLR7 signaling cascade (26, 28, 31). Although this sequence of events ensures specificity and safety, it is likely more susceptible to various regulatory mechanisms.

Lymphocytic choriomeningitis virus Cl13 stimulates immune cells not only through the TLR7/MyD88 pathway (15, 16) but also through the RIG-I/Mda5 pathway (47). In order to further study the mechanisms at play in this setting and isolate the TLR7 pathway from other variables of the LCMV infection, we pretreated mice with PapMV followed by a second immunization at various time points. This approach recapitulated the results observed in LCMV-infected mice with almost complete abrogation of IFN-α production and pDC activation following the secondary PapMV immunization for short time intervals between immunizations and significant impairment for longer intervals (Figure 2). A similar outcome was observed for the production of various cytokines and chemokines such as TNF-α, IL-6, IL-12p40, IL-12p70, IL-15, M-CSF, RANTES, and IP-10 while others were either not affected or enhanced by the secondary immunization (Figure S1 in Supplementary Material). This suggests that PapMV stimulates other pathways in addition to TLR7 leading to a broad activation of the immune system and that these pathways might be differently affected by multiple PapMV administrations. Nonetheless, the main outcome of multiple systemic administrations of PapMV, at least for the TLR7 pathway, is the suppression of the secondary response. This outcome was also observed in previous studies and is indicative of TLR tolerance (discussed below) (50, 52–54). We posit that the inability of subsequent PapMV immunizations to drive a robust response may also be dependent on the route of administration. This conclusion comes from our previous findings showing that sequential intranasal instillations could potentiate PapMV treatments (30). In the previous study, immunizations were separated by seven days and the last immunization led to a higher production of various cytokines in bronchoalveolar lavages. In an intratumoral injection model, we also observed that multiple administrations of PapMV led to decreased tumor growth when administered alone or in combination with other immunotherapies and sustained IFN-α following multiple administrations (37). Pretreatment with PapMV is therefore able to potentiate further PapMV administrations when delivered locally. Limitations are however observed when PapMV is administered systemically, as shown in this study. The development of immunization regimens alternating between various administration routes could therefore be an interesting alternative to mitigate the pitfall of sequential systemic treatments.

With regard to the tolerization of TLRs, we found that degradation of IRAK1 played a central role. This is in agreement with other studies illustrating that this kinase, which is shared across most of the TLR pathways (56), is degraded following TLR2, TLR4, TLR7 and TLR9 stimulation (52, 55, 69, 70). Although this degradation has been shown to last at least 48 h poststimulation (54, 55) both *in vivo* and *in vitro*, the length of this refractory period has yet to be determined. Here, we show that the stimulation of BMpDCs with PapMV induces a partial degradation of IRAK1, which was observed by flow cytometry and later confirmed by immunoblotting (Figure 3). Of note, we observed a stronger IRAK1 degradation with R837 than with PapMV, which might be due to the different nature of both TLR7 agonists. Indeed, R837 is a small synthetic molecule that does not require uptake to reach the endosome of cells. It is therefore easier and faster for this molecule to reach more cells and induce the degradation of IRAK1 in a more robust fashion. On the other hand, PapMV is a particulate molecule that has to be taken up by immune cells to reach the endosome thus elongating the interval between the stimulation and the apparent degradation of IRAK1 (71). It would also be of particular interest to verify the regulation of IRAK1 in pDCs *in vivo*. However, due to the low proportion of pDCs in the spleen, we were limited to conducting our analyses *in vitro* to determine the regulation of IRAK1 in BMpDCs. It is important to note that our results revealed only a partial role played by IRAK1 in the tolerance observed following multiple administrations of PapMV. Indeed, stimulation of BMpDCs with PapMV did not induce complete degradation of IRAK1, suggesting that there could be residual proteins left in the cells capable of proceeding through the signaling cascade when further encountering PapMV. This led us to investigate other potential inhibitory mechanisms.

Niederquell et al. suggested that expression of Sca-1 could discriminate between subsets of pDCs able or not to produce IFN-α in response to TLR stimulation (53). We therefore hypothesized that sequential PapMV administrations could preferentially stimulate or expand pDC subsets unable to produce IFN-α, which might explain the abrogation of IFN-α production upon secondary immunizations. However, in our system, Sca-1 expression on pDCs was not associated with the capacity to produce IFN-α in response to PapMV. While we used a particulate molecule, Niederquell et al. used CpG ODN as a TLR9 ligand. Given that TLR7 and TLR9 are not stimulated by the same ligands (RNA vs. DNA) and similar to R837, CpG ODN is a small synthetic molecule, the kinetics of activation are therefore different in both models. In this regard, other markers such as Ly49Q (57), CD123 (72), and CD9 (59) that have also been associated with IFN-α production might be more informative.

Administration of plant virus-like particles in mice leads to the rapid production of specific antibodies [reviewed in Ref. (73)]. Since antibodies are generated following PapMV injection, we were interested in assessing their impact on multiple administration regimens. We showed by immunizing J_H_T mice, which are devoid of B cells and antibodies, as well as performing serum transfer, that PapMV-specific antibodies generated after the first administration were largely responsible for the tolerance to a second PapMV injection. In the short-term immunization regimen (5 days), there was retained inhibition of pDC activation in J_H_T mice as shown by the slightly diminished expression of CD69 following a second immunization relative to the naive group (Figure 5B). When transferring PapMV immune serum from day 5 postimmunization into naive C57Bl/6 mice followed by PapMV immunization, we observed lower expression levels of CD69 and CD86 compared to mice receiving a single PapMV administration (Figure 5F compared to Figure 2B). This is either due to an underlying mechanism independent of antibodies or due to the titer of antibody transferred. Although the titer of PapMV specific antibodies found in mice receiving serum transfers is lower than what is found in PapMV immunized mice (Figure S3 in Supplementary Material), this was enough to interfere with the subsequent PapMV immunization by inhibiting the production of IFN-α on both days 5 and 25. To overcome this limitation, one could administer immunogenically distinct plant virus-like particles to circumvent the effect of antibodies. It would also be interesting to explore the use of different injection routes and whether or not antibodies can also interfere with the response to subsequent injections.

Other directions are currently being assessed to potentiate systemically administered PapMV. We have previously shown that our platform could be modified in order to display various epitopes on the surface of the nanoparticle (25, 27, 28, 33, 74–76). These engineered particles showed immunostimulatory properties similar and sometimes better than the original platform following immunization (25, 27, 28, 33, 74, 76). A new strategy that we are currently investigating is the use of a sortase-mediated antigen coupling technique, which permits the fusion of epitopes directly on the surface of PapMV without the need to genetically modify the sequence of the coat protein (36). Immunizations with such PapMV-fused platforms lead to the development of protective humoral responses (36). Different immunization regimen as well as different chronic viral infection models could here be tested to evaluate the potential of PapMV in treating other diseases. The results obtained during this study open the way to study other potential uses for PapMV such as in the treatment of autoimmune diseases. It was previously shown that in the absence of IFN-α, whether in IFNAR-deficient mice or through the use of IFNAR antibody blockade, autoimmune symptoms of lupus prone mice were improved (77, 78). Multiple systemic administrations of PapMV induced an inhibition of IFN-α production providing a potential therapeutic approach for such an application.

In this study, we showed that treatment of a chronic virus infection with PapMV has limitations and still needs to be improved. Although a single administration of PapMV induces strong immune responses, recall systemic immunizations are much less potent partly due to IRAK1 degradation but mainly to interference by PapMV-specific antibodies. Our results also demonstrate that the PapMV platform is able to induce an immune response following a pretreatment although not yet to a degree that would be able to clear an ongoing viral infection. Although this outcome is not favorable in the context of chronic viral infections, it could be of interest for other diseases such as autoimmunity. Further improvements will therefore be needed for this promising therapeutic approach to be used in the treatment of chronic viral infections or other IFN-dependent chronic diseases.

## Ethics Statement

This study was carried out in accordance with the recommendations of the Canadian Council on Animal Care guidelines. The protocol was approved by the Institut national de la recherche scientifique Animal Care Committee.

## Author Contributions

KC, M-ÈL, and AL conceived and designed the experiments; KC, M-ÈL, and ET performed the experiments; PS developed the PapMV manufacturing process; KC, AL, and DL cowrote the article.

## Conflict of Interest Statement

DL is the founder and a shareholder of Folia Biotech. Inc., a Canadian Biotechnology Company with the mandate to commercialize the PapMV Technology. The other authors have no financial conflicts of interest.

## Supplementary Material

Figure S1 Multiplex quantification of cytokines and chemokines in serum 6 h following the last immunization with PapMV. Immunizations were performed 7 or 14 days following a first immunization with PapMV (*n* = 1, five mice per group).

Figure S2 CD69 expression kinetics on plasmacytoid dendritic cells after PapMV immunization. Results are presented as a ratio of the MFI of the sample over the average mean fluorescence intensity of controls (*n* = 1–7, one to three mice per group).

Figure S3 ELISA quantification of PapMV-specific IgG in serum of mice transferred with immune sera 6 h following an immunization with control or PapMV. Immune sera were collected 5 and 25 days following PapMV immunization (*n* = 2, two to three mice per group).

## Abbreviations

BFA, Brefeldin A; BMpDCs, bone marrow-derived plasmacytoid dendritic cells; dpi, days postinfection; FBS, fetal bovine serum; HBV, hepatitis B virus; HCV, hepatitis C virus; IFN, interferon; IRAK1, interleukin-1 receptor-associated kinase 1; LCMV Cl13, lymphocytic choriomeningitis virus clone 13; LPS, lipopolysaccharide; MFI, mean fluorescence intensity; MHC-I, class I major histocompatibility complex; PapMV, papaya mosaic virus nanoparticle; pDCs, plasmacytoid dendritic cells; Poly I:C, polyinosinic:polycyticylic acid; TLR, toll-like receptor; WT, wild type.
