# Supplementary figures and images for: Efficacy of a Virus-Like Nanoparticle As Treatment for a Chronic Viral Infection Is Hindered by IRAK1 Regulation and Antibody Interference
Source: Front Immunol. 2018 Jan 4;8:1885. doi: 10.3389/fimmu.2017.01885 (PMC5758502; doi:10.3389/fimmu.2017.01885)

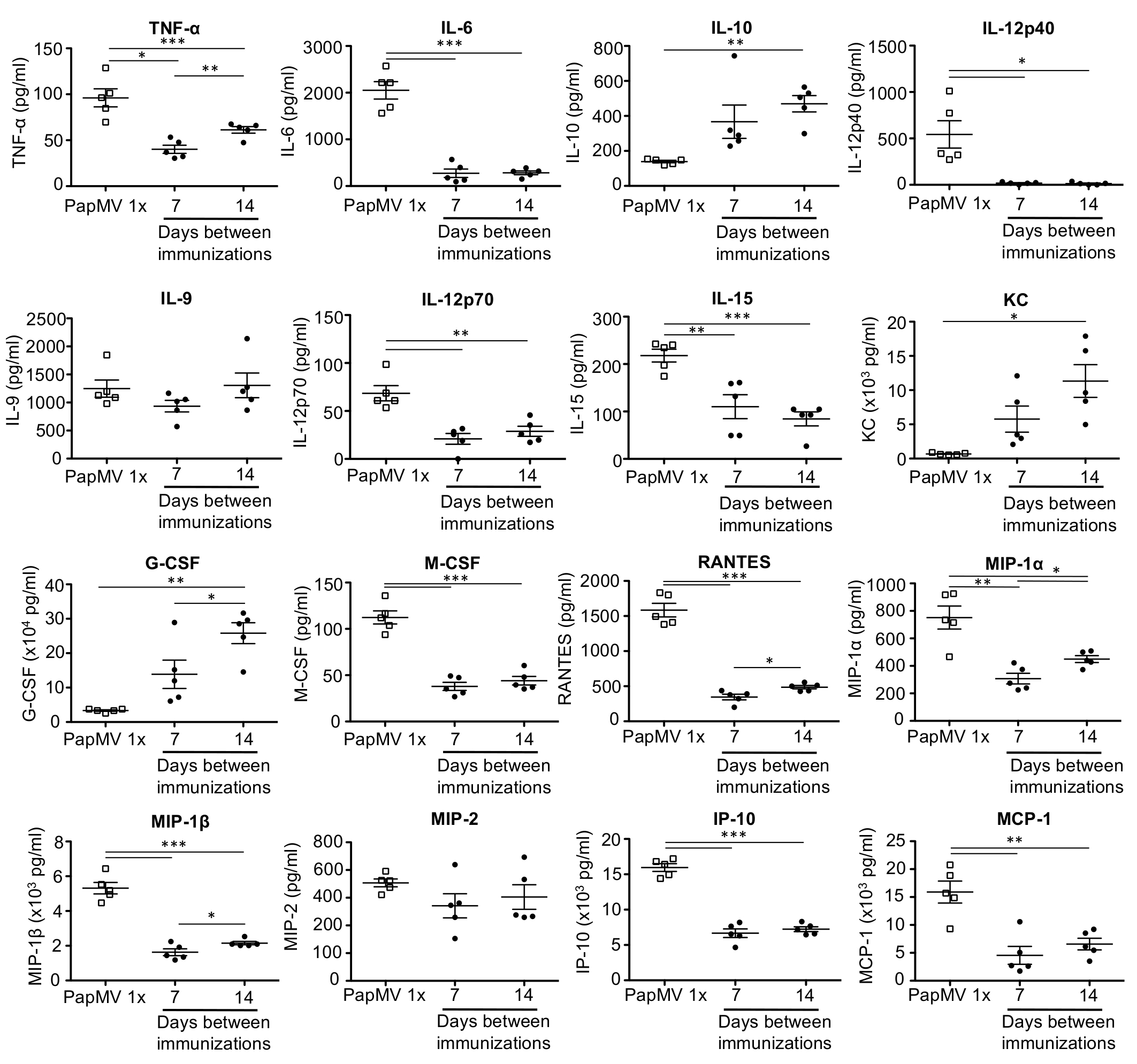

Supplement: Figure S1 — Multiplex quantification of cytokines and chemokines in serum 6 h following the last immunization with PapMV. Immunizations were performed 7 or 14 days following a first immunization with PapMV (n = 1, five mice per group). [file Image_1.jpeg]

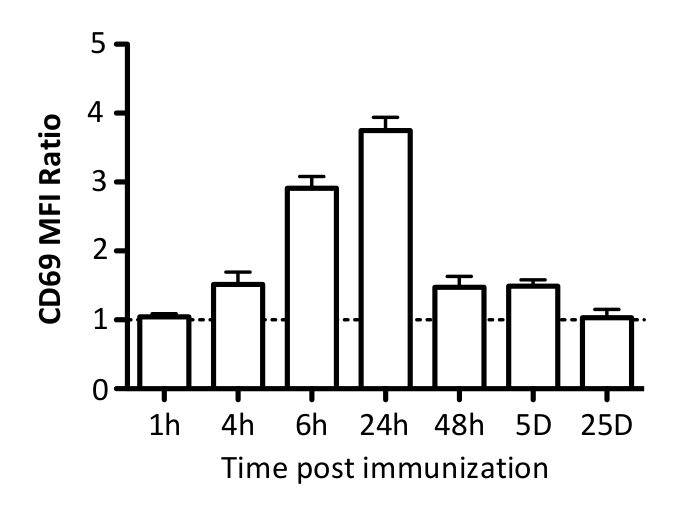

Supplement: Figure S2 — CD69 expression kinetics on plasmacytoid dendritic cells after PapMV immunization. Results are presented as a ratio of the MFI of the sample over the average mean fluorescence intensity of controls (n = 1–7, one to three mice per group). [file Image_2.jpeg]

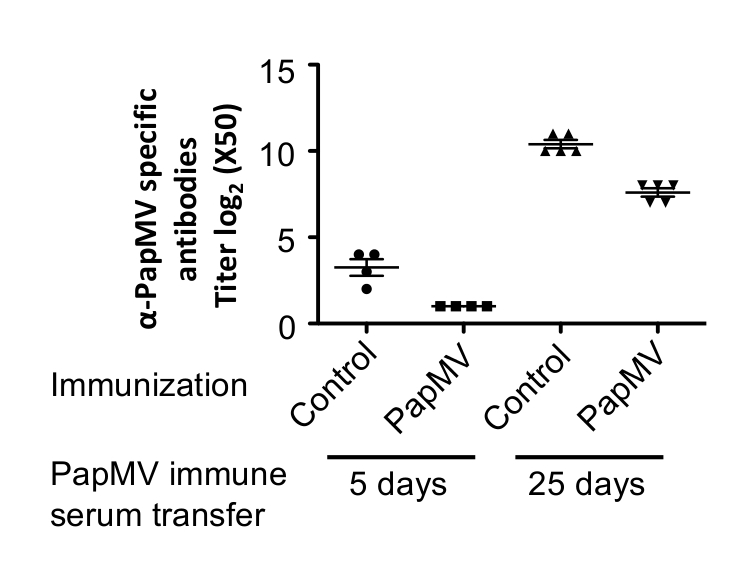

Supplement: Figure S3 — ELISA quantification of PapMV-specific IgG in serum of mice transferred with immune sera 6 h following an immunization with control or PapMV. Immune sera were collected 5 and 25 days following PapMV immunization (n = 2, two to three mice per group). [file Image_3.jpeg]
